# Biological variation of thyroid stimulating hormone, free triiodothyronine and free thyroxine in healthy subjects in Turkey

**DOI:** 10.11613/BM.2025.010706

**Published:** 2025-02-15

**Authors:** Raziye Yıldız, Hayat Özkanay, Fatma Demet Arslan, Mehmet Köseoğlu

**Affiliations:** 1 Department of Medical Biochemistry, Bakırcay University, Izmir, Turkey; 2 Department of Medical Biochemistry, Katip Celebi University, Izmir, Turkey

**Keywords:** thyroid hormones, biological variation, healthy people, Turkey

## Abstract

**Introduction:**

Biological variation (BV) data are necessary for interpretation of test results and assessment of analytical performance. We aimed to determine the BV estimates for thyroid stimulating hormone (TSH), free triiodothyronine (fT3) and free thyroxine(fT4) in healthy subjects in Turkey and compare them with the literature findings.

**Materials and methods:**

A total of 21 Turkish healthy volunteers (12 males and 9 females) were included in the study. Blood samples were collected once a week for five weeks, and the analysis was performed using the chemiluminescent immunoassay method on an Advia Centaur XP (Siemens Diagnostic, Tarrytown, USA). Analytical variation (CV_A_), within-subject BV (CV_I_) and between-subject BV (CV_G_) were calculated. Analytical goals, individuality index (II) and reference change value (RCV) were derived from these data. Statistical analysis was performed using BioVar: BV analysis tool v.1.0.

**Results:**

For TSH, fT3 and fT4, CV_A_ (confidence interval, CI) were 3.3% (2.9 to 3.8), 1.7% (1.5 to 1.9) and 2.7% (2.4 to 3.1); CV_I_ (CI) were 22.3% (19.3 to 26.3), 4.4% (3.8 to 5.3) and 5.1% (4.3 to 6.1); CV_G_ (CI) were 26.6% (19.2 to 39.8), 9.2% (6.9 to 13.6) and 8.2% (6.1 to 12.1), respectively. For TSH, fT3 and fT4, desirable total errors were 27.1%, 6.2% and 6.6%; II values were calculated as 0.84, 0.48 and 0.61; and RCV% values (decrease; increase) were - 40.3;67.6, - 10.4;11.6 and - 12.7;14.5, respectively.

**Conclusions:**

Our study provides updated BV data for thyroid function tests (TFTs) in healthy subjects in Turkey. As TFTs have shown a high degree of individuality, RCV should be preferred rather than population-based reference ranges in the assessment of serum concentrations. Our BV estimates were compatible with European Federation of Clinical Chemistry and Laboratory Medicine (EFLM) BV meta-analysis data obtained using different immunoassay methods in different populations.

## Graphical abstract

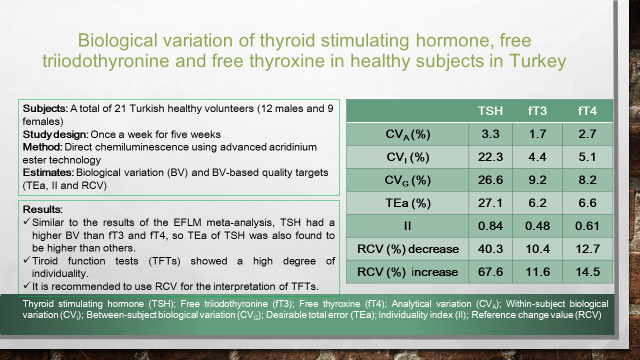

## Highlights

• This study provided updated biological variation (BV) estimates for thyroid hormones in 21 healthy Turkish volunteers

• Although BV estimates for thyroid function tests in our study were found to be different than some previously published, they were compatible with European Federation of Clinical Chemistry and Laboratory Medicine BV meta-analysis data obtained using different immunoassay methods in different populations

• The BV estimates and analytical performance specifications of thyroid stimulating hormone were higher than those of free triiodothyronine and free thyroxine

• As thyroid hormones have low individuality index, reference change value should be preferred over the population-based reference interval to interpret of test results

## Introduction

The prevalence of spontaneous hypothyroidism is 1-2%, and the prevalence of hyperthyroidism is 0.5-2% and it is more common in women than in men. It is suggested that if healthy adults were screened for thyroid disease, the prevalence of subclinical hypothyroidism and subclinical hyperthyroidism could be approximately 10% and 1%, respectively (*1*). The clinical symptoms and signs of thyroid disorders are usually nonspecific and progress slowly. The correct interpretation of laboratory tests is important in the diagnosis and follow-up of thyroid diseases. Current guidelines recommend using thyroid stimulating hormone (TSH) as the first step to detect thyroid dysfunction because of the log-linear relationship of TSH with free thyroxine (fT4). Free thyroxine and, in some clinical situations, free triiodothyronine (fT3) are added as a second step when TSH concentrations are outside the reference range, these tests allow thyroid disease to be classified as overt or subclinical (*2*).

Population-based reference ranges for thyroid function tests (TFTs) are quite large due to differences in preanalytical, analytical, and biological variations (*3*). Interpretation of test results based solely on reference ranges may mask clinically important differences because TFTs have narrow intra-individual variability (*4*).

Biological variation (BV) data facilitates clinical decision-making by helping to correctly interpret test results. The BV has two components: within-subject variation (CV_I_) and between-subject variation (CV_G_). The BV estimates are used to the calculation of reference change value (RCV) and individuality index (II). When the individuality of an analyte is high, the usefulness of population-based reference intervals is limited, and in this case the use of RCV is recommended (*5*). The BV is also important for defining analytical performance specifications (APS) (imprecision and bias) (*5*).

Some studies on the BVs of TSH, fT3, and fT4 measured with different methods have been reported in different populations (*6*-*11*). In a recent study from Turkey, it was evaluated the clinical significance of indirect reference intervals by use of RCV of the TFTs (*12*). Reference change value was calculated using analytical variation (CV_A_) value obtained by the electrochemuliminescent method in their laboratory and CV_I_ value taken from the European Federation of Clinical Chemistry and Laboratory Medicine (EFLM) database in this study (*12*). To the best of our knowledge, there has not been CV_I_ and CV_G_ data obtained by the chemuliminescent method in the Turkish population.

We aimed to find out whether the BV estimates of TSH, fT3 and free fT4 tests vary in different populations and with different analytical methods, and to determine the APS, RCV, and individuality index (II) values from our BV data.

## Materials and methods

### Subjects

The study was carried out in the Medical Biochemistry Laboratory of İzmir Katip Çelebi University, Atatürk Training and Research Hospital. A total of 21 healthy volunteers, which are 12 males and 9 females, were included in this study. Our study was conducted in March and April 2020.

Subjects were selected in accordance with the inclusion/exclusion criteria of the Biological Variation Working Group (*13*). Participants’ medical histories were obtained before inclusion in the study. Participants were over 18 years old, in good health, non-smoking, not taking medications, out of strenuous exercise, had no illness at the time of the study, and had no thyroid diseases or a family history of thyroid diseases. The women weren’t pregnant or in lactation and had regular menstrual cycles. In addition, routine blood tests of subjects such as complete blood count, glucose, creatinine, blood urea nitrogen, gamma-glutamyl transferase, alanine aminotransferase, aspartate aminotransferase, total cholesterol, triglycerides, ferritin and C-reactive protein were performed to check their routine control before inclusion in the study. Their test results were within reference ranges. During the study, all subjects maintained their usual lifestyles.

This study was conducted in accordance with national regulations and Helsinki Declaration (as revised in 2013). Additionally, the written informed consent was obtained from the subjects. This study was approved by the Local Ethics Committee of Katip Çelebi University (Reference number:44/2020).

### Methods

The protocol of our study followed checklist produced by the EFLM Biological Variation Working Group (WG-BV) (*14*). Venous blood samples were collected on the same days for five consecutive weeks. All samples were drawn between 8:00 and 10:00 a.m. Participants were fasted for at least 8-12 hours and kept in a sitting position for at least 5 minutes *prior* to blood collection (*13*). A blood collection tubes containing clot activator and gel separator (BD Vacutainer SST-II Advance, Plymouth, UK) were used. Venous blood samples were centrifuged at 1500×g for 10 minutes after coagulation. The separated sera were aliquoted and stored for 2 months at - 80 °C until analyzed. Samples were thawed for 30 min at room temperature and mixed thoroughly before analysis.

All samples were tested in duplicate in the same run. The fT3, fT4, and TSH were measured by direct chemiluminescence immunoassay (CMIA) using advanced acridinium ester technology on an Advia Centaur XP immunoassay analyzer (Siemens Diagnostic, Tarrytown, USA) with the Advia Centaur TSH, fT3 and fT4 reagents.

To provide the quality assurance of tests, Lyphochek Immunoassay Plus Control Trilevel 370 (Bio-Rad Laboratories Inc., San Diego, USA) was used as internal quality control material and EQAS Immunassay (Monthly) Program BC75 (Bio-Rad Laboratories Inc., San Diego, USA) as external quality control material.

### Statistical analysis

Statistical analysis for the calculation was performed using version 14.0 Microsoft Excel and BioVar web tool for BV analysis (*15*). BioVar web tool calculates BV in seven steps. Firstly, it identifies outliers using Cochran’s test and Reed’s criterion. Secondly, the normal distribution is checked using the Shapiro-Wilk test. In third step, a linear regression analysis is used to assess whether the concentrations of analyte during the study period are in steady state. In the fourth step, the homogeneity of CV_A_ and CV_I_ are determined by the Bartlett test. In fifth step, a subgroup analysis based on the overlap of 95% confidence intervals (CIs) of BV between gender is performed. Finally, CV_A_, CV_I_ and CV_G_ are calculated using a two-fold nested ANOVA. The BV values are given as a percentage. Differences in body mass index (BMI) and age between men and women were analyzed using the Mann-Whitney U test, and differences in test results were analyzed using the Student t-test. P < 0.05 values were considered statistically significant.

The II for each analyte was calculated from CV_I_ and CV_G_ using the formula: II = CV_I_ / CV_G_. The RCV (decrease; increase) were determined by the calculation tool in EFLM-BV web site (*16*). We calculated minimum, desirable and optimal analytical goals for imprecision%, bias% and total error (TE%) by using the tool in EFLM-BV web site (*16*).

## Results

The median (range) of age of male (N = 12) and female (N = 9) were 39 (28-55) and 34 (27-43) (P = 0.046), the median (range) of BMI were 26 (21-30) and 23 (18-29) kg/m^2^ (P = 0.030), respectively.

There were 105 samples and 210 data for each test. The outliers in the replicate analyses were detected and two duplicate data were removed for both fT4 and TSH. The BVs were estimated with a total of 208 data for both fT4 and TSH. As the distribution of fT3 data in one male was wider than in all groups, according to the criteria incorporated in BioVar: BV analysis tool v.1.0 (*15*), these data (N = 10) were removed. The BV was estimated with a total of 200 data for fT3.

The mean of fT4 results of subjects had a non-normal distribution. To ensure normal distribution, the BV of fT4 was calculated after the back-log transformation of data.

All subjects were in a steady state for fT3 and fT4, except TSH. After the multiples of median transformation of TSH data, it became a stable state. The CV_A_ and CV_I_ of the three parameters were shown homogeneity.

**Figure 1:**
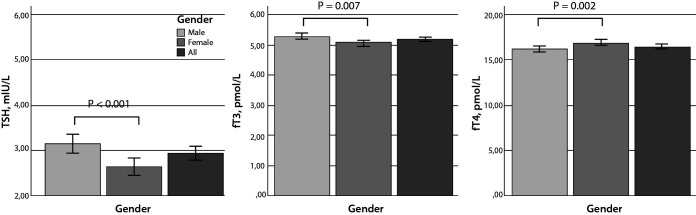
The comparisons of mean (Cl 95%) of thyroid function tests between males and females. TSH - thyroid stimulating hormone. fT3 - free triiodothyronine. fT4 - free thyroxine.

The mean (95% CI) of TSH, fT3 and fT4 results in males, females and all individuals are shown in Figure 1. There was a difference between males and females for all parameters (TSH, fT3 and fT4).

In our study, the CV_A_ (95% CI) for TSH, fT3 and fT4 were calculated 3.0% (2.5-3.6), 1.7% (1.4-2.1) and 2.9% (2.4-3.5) in males and 3.7% (3.1-4.7), 1.6% (1.4-2.1), and 2.5% (2.0-3.1) in females, respectively. The CV_A_ values for all samples were shown in Table 2.

**Table 2:** Analytical and biological variation values with 95% CI for all participants, RCV and II values

**Study (year of publication, reference)**	**CV_I_ (%)**			**CV_G_ (%)**			**CV_A_ (%)**	**RCV (%)**	**II**
	**Female**	**Male**	**All**	**Female**	**Male**	**All**	**All**	**Decrease; Increase**	
TSH									
Our study	22.6 (18.3-29.6)	22.0 (18.3-27.5)	22.3 (19.3-26.3)	18.8 (9.6-39.7)	28.1 (18.5-49.6)	26.6 (19.2-39.8)	3.3 (2.9-3.8)	- 40.3; 67.6	0.84
Meta-analysis of EFLM (16)*	NA	NA	17.9 (14.7-29.3)	NA	NA	36.1 (23.9-48.4)	NA	NA	0.50
Bottani M (2021, (7))	16.8 (15.7-18.1)	18.9 (17.5-20.5)	17.7 (16.8-18.7)	46.3 (38.9-62.1)	35.9 (28.9-48.0)	35.9 (31.1-42.4)	1.5 (1.4-1.5)	- 33.6; 50.7	0.49
Mairesse A (2021, (11))	NA	NA	19.7 (17.0-23.5)	NA	NA	37.6 (28.0-57.1)	0.9 (0.8-1.1)	- 36.4; 57.3	0.52
Ankrah-Tetteh T (2008, (8))	NA	NA	25.1 (0.0-32.8)	NA	NA	36.9 (24.3-66.8)	13.5	- 47.9; 92.0	0.68
Maes M (1997, (6))	NA	NA	29.3 (27.0-32.0)	NA	NA	48.4 (37.7-66.8)	5.8	- 49.3; 97.2	0.60
Polo MJ (1992, (10))	NA	NA	20.6	NA	NA	29.4	NA	NA	0.70
Ricos C (1990, (9))	NA	NA	19.3 (16.8-22.7)	NA	NA	19.7 (13.6-31.7)	4.7	- 36.7; 57.9	0.97
**fT3**									
Our study	5.3 (4.2-6.9)	3.7 (3.0-4.8)	4.4 (3.8-5.3)	9.2 (5.9-18.0)	9.2 (6.3-16.3)	9.2 (6.9-13.6)	1.7 (1.5-1.9)	- 10.4; 11.6	0.48
Meta-analysis of EFLM (16)*	NA	NA	5.1 (4.7-7.9)	NA	NA	8.1 (8.0-22.5)	NA	NA	0.63
Bottani M (2021, (7))	5.3 (5.0-5.7)	4.6 (4.3-5.0)	5.0 (4.7-5.3)	9.8 (8.0-12.3)	8.0 (6.5-10.5)	8.0 (6.9-9.5)	1.8 (1.7-1.8)	- 11.6; 13.1	0.62
Mairesse A (2021, (11))	NA	NA	6.0 (5.1-7.2)	NA	NA	8.6 (6.3-13.3)	2.2 (1.9-2.5)	- 13.8; 16.0	0.7
Ankrah-Tetteh T (2008, (8))	NA	NA	4.7 (3.7-5.9)	NA	NA	12.7 (8.7-22.5)	1.2	- 10.6; 11.9	0.37
**fT4**									
Our study	5.1 (4.0-6.8)	5.0 (4.0-6.5)	5.1 (4.3-6.1)	6.6 (4.0-13.3)	9.1 (6.2-15.9)	8.2 (6.1-12.1)	2.7 (2.4-3.1)	- 12.7; 14.5	0.61
Meta-analysis of EFLM (16)*	NA	NA	4.8 (4.8-9.5)	NA	NA	8.0 (7.5-12.1)	NA	NA	0.60
Bottani M (2021, (7))	5.0 (4.6-5.3)	4.5 (4.2-4.9)	4.8 (4.5-5.1)	10.2 (8.4-12.7)	7.5 (6.1-9.9)	7.5 (6.5-8.9)	1.7 (1.6-1.8)	- 11.2; 12.6	0.64
Mairesse A (2021, (11))	NA	NA	4.6 (3.8-5.8)	NA	NA	10.8 (8.0-16.3)	3.6 (3.2-4.2)	- 12.7; 14.5	0.43
Ankrah-Tetteh T (2008, (8))	NA	NA	4.8 (1.4-6.2)	NA	NA	11.7 (8.0-20.8)	2.4	- 11.9; 13.4	0.41
Maes M (1997, (6))	NA	NA	7.1 (3.0-8.8)	NA	NA	9.1 (6.9-12.8)	7.5	- 21.3; 27.0	0.78
Polo MJ (1992, (10))	NA	NA	5.6	NA	NA	12.3	NA	NA	0.45
*BV meta-analysis data on the EFLM website (16). TSH - thyroid stimulating hormone. fT3 - free triiodothyronine. fT4 - free thyroxine. CV_A_ - analytical variation. CV_I_ - within-subject biological variation. CV_G_ - between-subject biological variation. RCV - reference change value. II - individuality index. 95% CI - confidence interval of 95%. NA - not available.									

The features and BV data from other studies and our study, and BV meta-analysis data on the EFLM-BV website were shown in Table 1 and 2 (*16*). The CV_I_ and CV_G_ of fT3 and fT4 were lower than those of TSH in all studies including ours. In our study, the CV_A_, CV_I_ and CV_G_ values were not found to be different between gender as their CIs overlap.

**Table 1:** The characteristics of all studies including our study

**Study (year of publication, reference)**	Score*	**Included in the EFLM meta-analysis**	**Method (manufacturer)**	**Sampling time**	**Male/Female**
Our study	A_1,2,3,4,5,6,7,8,9,10,11,12,13,14_	No	CMIA (Siemens)	1 *per* week, 5 weeks	12/9
Bottani M (2021, (7))	A_1,2,3,4,5,6,7,8,9,10,11,12,13,14_	Yes	ECLIA (Roche)	1 *per* week, 10 weeks	38/53
Mairesse A (2021, (11))	B_7_	Yes	ECLIA (Roche)	1 *per* week, 5 weeks	8/11
Ankrah-Tetteh T (2008, (8))	C_7,8,10,13_	Yes	CMIA (Siemens)	1 *per* week, 6 weeks	4/6
Maes M (1997, (6))	C_8,10,11_	Yes	IRMA (Baxter) for TSH ELISA (Abbott) for fT4	1 *per* month, 1 year	13/13
Polo MJ (1992, (10))	C_12_	No	NA	1 *per* day, 5 days	12/13
Ricos C (1990, (9))	C_4,8,10_	No	ELISA (Boehringer Mannheim)	1 *per* day, 1 week	6/9
*Biological Variation Data Critical Appraisal Checklist based on 14 quality indicators (IQs) is scored as A, B, C, and D (17). The IQs are related to scale of measurand, subjects, samples, measurement procedure, preanalytical procedure, the estimate of analytical variation, steady state of individuals, outliers, distribution of data, variance homogeneity, statistical method, confidence intervals, number of included results and concentrations of measurand. An A score indicates full compliance with all the 14 BIVAC QIs. The estimates from a study receiving any D score are unsuitable for clinical application. If the lowest QI score is the publication score. The QIs associated with the A-D scores are given as a subscript. TSH - thyroid stimulating hormone. fT4 - free thyroxine. CMIA - chemiluminescence immunoassay. ECLIA - electrochemiluminescence immunoassay. IRMA - immunoradiometric. ELISA -enzyme-linked immunosorbent assay					

While the fT3 II value was below 0.6 (which is exhibited low II), fT4 and TSH II values were found to be between 0.6-1.4 (Table 2) (*5*). The highest RCV% limits were determined for TSH (Table 2). The APS such as imprecision%, bias% and TE% derived from the BV data are shown in Table 3. The APS values of fT3 and fT4 were narrower than those of TSH in our study.

**Table 3:** Analytical performance specifications at minimum, desirable and optimal levels

**Parameter**	**Imprecision%**			**Bias%**			**Total error%**		
	**M**	**D**	**O**	**M**	**D**	**O**	**M**	**D**	**O**
TSH	16.7	11.2	5.6	13.0	8.7	4.3	40.6	27.1	13.5
fT3	3.3	2.2	1.1	3.8	2.5	1.3	9.3	6.2	3.1
fT4	3.8	2.5	1.3	3.6	2.4	1.2	9.9	6.6	3.3
M - minimum. D - desirable. O - optimal. TSH - thyroid stimulating hormone. fT3 - free triiodothyronine. fT4 - free thyroxine.									

## Discussion

Our results showed that TSH has a larger BV than fT3 and fT4 in Turkish healthy individuals, and the RCV usage would be more suitable in TFTs results interpretation.

Biological Variation Working Group and Task and Finish Group published the Biological Variation Data Critical Appraisal Checklist (BIVAC), which includes A, B, C and D scores based on 14 quality indicators (IQs) (*17*, *18*). The IQs are related to scale of measurand, subjects, samples, measurement procedure, preanalytical procedure, the estimate of analytical variation, steady state of individuals, outliers, distribution of data, variance homogeneity, statistical method, confidence intervals, number of included results and concentrations of measurand. An A score indicates full compliance with all the 14 BIVAC QIs. The estimates from a study receiving any D score are unsuitable for clinical application. For example, if the lowest QI score obtained is a B or C, the publication score is a B or C, respectively. The QIs associated with the A-D scores are given as a subscript. The use of BV has become safer for clinical practice by including BIVAC-compliant standardized studies in the EFLM-BV database (*16*). It was assumed that the total score of our BV study may be A_1,2,3,4,5,6,7,8,9,10,11,12,13,14_ according to BIVAC.

The estimate of BV of an analyte can be affected by many factors such as the population studied, age, gender, disease status, analytical method, timing of sampling and duration of the study (*5*). Both CV_I_ and CV_G_ values in our study were within the CI of EFLM meta-analysis data. In the literature, while the CV_I_ value of TSH was higher than in the study of Bottani *et al.* and lower than in the study of Maes *et al.*, the CV_G_ values for all tests in our study were not found to be different from other studies (*6*, *7*).

The CV_A_ of TSH analyzed by ECLIA method were 1.5% and 0.9%, by enzyme-linked immunosorbent assay (ELISA) method 4.7% and by immunoradiometric assay (IRMA) method 5.8% (*6*, *7*, *9*, *11*). The CV_A_ of TSH analyzed by the CMIA method was 3.3% in our study and 13.5% in another study (Table 2) (*8*). The CV_A_ of fT3 analyzed by the ECLIA methods were 1.8% and 2.2%, by CMIA methods 1.7% in our study and 1.2% in another study (Table 2) (*7*, *8*, *11*). The CV_A_ values of fT4 analyzed by CMIA methods (ours) were 2.7% and 2.4%, by ECLIA methods 1.7% and 3.6% and by ELISA method was 7.5% (Table 2) (*6*-*8*, *11*). Due to the contribution of analytical variation to the RCV, methodological differences should be taken into consideration and the standardization of analytical methods and TFT performance should be improved.

According to our study, while the BV of TFTs was compatible with those in the meta-analysis of EFLM, the II of it was found different from those in the meta-analysis of EFLM (*16*). In our population, while the usefulness of population-based reference intervals may be limited as the II of fT4 and TSH ranges from 0.6 to 1.4, it may be better to use RCV as the II of fT3 is < 0.6 (*5*, *18*).

The widest RCV range - 49.3% to 97.2% for TSH was observed in the Maes *et al.*’s study (Table 2) (*6*). These wide ranges for TSH may be due to high variations in CV_A_ and CV_I_. The widest RCVs of fT3 and fT4 were - 13.8% to 16.0% and - 21.3% to 27.0%, respectively (*6*, *11*). The wide RCV values in these studies may be attributed to the fact that the standardization of BIVAC was not fully met or the old measurement methods such as IRMA, were used. Because the CV_I_ of TSH was higher than that of fT3 and fT4 in all studies including ours, the RCV of TSH was also wide (*6*-*11*).

In a survey, it was stated that only 3.5% of clinicians had knowledge about BV and did not use BV data or RCV to interpret test results (*19*). The use of BV and RCV ensures good patient management, so clinicians should be trained on the usefulness of BV data in clinical decision making.

Robust BV data play an important role in increasing reliability of measurement (*20*). EFLM meta-analysis, Clinical Laboratory Improvement Amendments (CLIA), Royal College of Pathologists of Australasia (RCPA), National Center for Clinical Laboratories (NCCL) and National Academy of Clinical Biochemistry (NACB) propose allowable limits for TE% (*16*, *21*-*23*). Our desirable TE% limit for TSH was similar to acceptance limits of international guidelines and proficiency test organizers mentioned above. In addition, as the BV estimates of TSH were higher than those of fT3 and fT4, the desirable APSs of TSH were found to be higher than those of other TFTs (Table 3). While the desirable limits for fT3 and fT4 obtained from our study and the EFLM-BV meta-analysis data were similar, these limits were found to be much lower than other acceptance limits. This may be due to the use of different models to determine APS (*21*).

Our study has several limitations. Firstly, the seasonal variation in TFT was not taken into account, for which comprehensive studies including different seasons can be performed (*24*). In addition, the 5-week period in our study was slightly shorter than the follow-up time (6 weeks) of TFT in adult patients with thyroid dysfunction (*25*). There were also difference between the age and BMI of the males and females. It would be better if these matched. However, no significant differences in CV_A_, CV_I_ and CV_G_ were observed between genders.

In Bottani *et al.*’s study, although no significant differences were observed for BV estimates between men and women, BV values were presented separately for genders (*7*). They stated that no differences were found for mean values and BV estimates between the two female subgroups (females below and above 50 years), therefore only results from the overall female group were reported. In Mairesse *et al.*’s study, there was no significant difference in mean values of TFTs between males and females allowing us the derivation of sex-independent CV_I_ and CV_G_ values (*11*). None of the other studies examined the differences between gender, age and BMI of the individuals in BV estimation (*6*, *8*-*10*). As a result, it was thought that the BV differences may be independent of the differences in mean concentration, gender, age and BMI for TFT.

## Conclusion

Although our BV estimates differed from BV data obtained using different method and population in some previously published, we found similar results to the EFLM BV meta-analysis. In addition, TSH BV estimates were found to be higher than fT3 and fT4 with corresponding high in RCV and APS. It is also suggested that RCV should be used in the interpretation of results due to the high individuality of TFTs.

## Data Availability

All data generated and analyzed in the presented study are included in this published article.
